# Rapid injection of lumbar dorsal root ganglia under direct vision: Relevant anatomy, protocol, and behaviors

**DOI:** 10.3389/fneur.2023.1138933

**Published:** 2023-04-11

**Authors:** Xiaoman Yuan, Siyi Han, Fengtian Zhao, Anne Manyande, Feng Gao, Jie Wang, Wen Zhang, Xuebi Tian

**Affiliations:** ^1^Department of Anesthesiology, Hubei Key Laboratory of Geriatric Anesthesia and Perioperative Brain Health, and Wuhan Clinical Research Center for Geriatric Anesthesia, Tongji Hospital, Tongji Medical College, Huazhong University of Science and Technology, Wuhan, China; ^2^School of Human and Social Sciences, University of West London, London, United Kingdom; ^3^State Key Laboratory of Magnetic Resonance and Atomic and Molecular Physics, Key Laboratory of Magnetic Resonance in Biological Systems, Wuhan Center for Magnetic Resonance, Wuhan Institute of Physics and Mathematics, Chinese Academy of Sciences, Wuhan, China

**Keywords:** DRG injection, dorsal root ganglia, intraganglionic injections, neuropathic pain, chronic pain, SNI

## Abstract

**Introduction:**

Dorsal root ganglia (DRG) are anatomically well-defined structures that contain all primary sensory neurons and are distension nodules of the dorsal root in the spinal cord near the medial surface of each foramen. Therefore, DRG is considered to be a desirable target for injection to manage chronic pain. But it presents a limitation in probing deep into it without *in vivo* injection technology.

**Methods:**

Here, we described a technique for administering intraganglionic injections of lumbar DRG under direct vision. We use partial osteotomy rather than laminectomy, which removes more bone, to preserve spinal structures while gaining adequate DRG access. To monitor the intraoperative progress of the DRG injection, a non-toxic dye was utilized. The effectiveness of the injection on the diffusion of AAV (adeno-associated virus) within the ganglion was assessed by histopathology at postoperative day 21.

**Results:**

Behavioral tests showed that neither motor nor sensory abilities were affected by saline or AAV injections. Meanwhile, the decreased pain threshold of SNI (spared nerve injury) was considerably restored by pharmacological inhibition of DRG neurons.

**Discussion:**

Our research achieved a new minimally invasive and intuitive intra-ganglionic injection in mice. In addition, the present protocol may serve as a valuable resource for planning preclinical studies of DRG injection.

## Introduction

**Figure 1:**
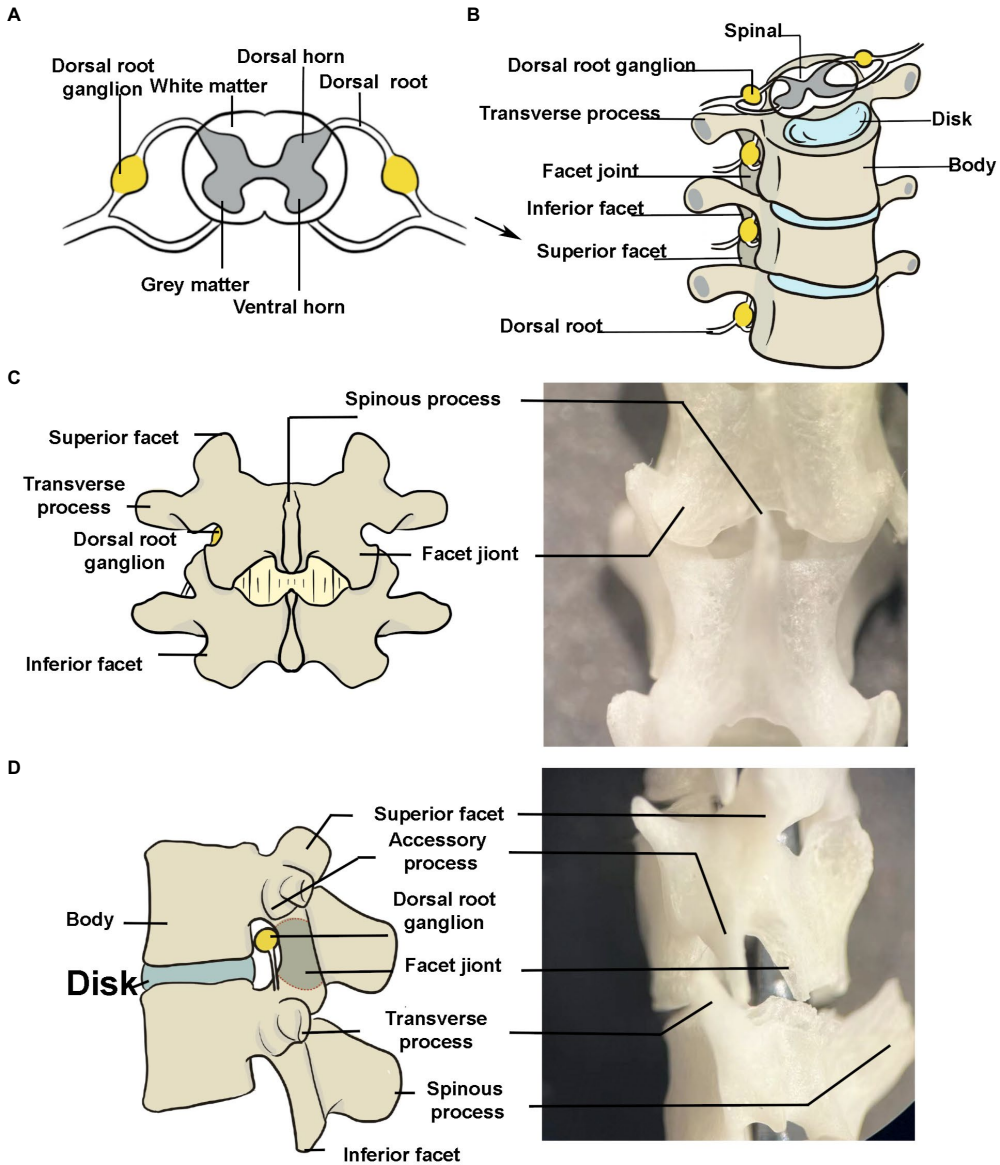
Peripheral anatomy of the dorsal root ganglion (DRG). **(A)** The DRG is a cluster of sensory neuron cell bodies located in the dorsal roots of the spine. The schematic portrays a transverse section of the spine. **(B)** The bony structures surrounding the DRG (lumbar segments, anterior view). The transverse process has been exaggerated to make it easier to understand. **(C,D)** Posterior and left side view of spine. The DRG is seen in the foramina. It is difficult to see the transverse processes in the posterior view due to the short transverse process in mice. The image depicts a large one for better understanding.

The dorsal root ganglion (DRG) is a bilateral structure that is bundle-shaped, entering dorsally from the spinal cord symmetrically from left to right within fixed bony vertebral structures (neuroforamen) (1, 2) (Figures 1A,B). At the segmental levels that innervate the limbs, each DRG has up to 15,000 neurons (3). The DRG, which is an extension of the dorsal root, is where primary sensory neurons’ somata are situated (4). The DRG is an essential structure for modulating and transducing sensory information, including the transmission of pain (1). Nerve injury-related pain is typically persistent and difficult to treat (5), and physiological modifications in the spinal cord and brain activity occur along with neuropathic pain (6). Meanwhile, numerous studies have demonstrated that damage to the primary sensory neurons and their somata in the DRG are significant sites for the development of pain (7–9). The DRG exhibits notable phenotypic and functional changes after injury, and these plastic alterations in the DRG as the primary source of pain signals delivered to the brain (7). Additionally, rodent DRG neurons have been used to investigate sensory nerve development, regeneration, and function to support medical research and explain the mechanism of peripheral nerve disorders such Charcot–Marie–Tooth disease and diabetic neuropathy (10, 11).

Direct injections into the DRG offer the chance to alter sensory neuron function at a segmental level in order to investigate the pathophysiology of nociceptive diseases. Intrathecal injection has been a widespread way to deliver medications to sensory neurons in recent years, but it is impossible to confine the solution to a certain longitudinal length on one side or at the vertebral level (12, 13). According to Moore et al.’ research, agents can be injected into peripheral nerves to reach the DRG, but it could injure the spinal cord and surrounding nerves and induce the agents to spread (14, 15). Manipulating the neuron of one or more DRGs can increase therapeutic effectiveness while minimizing side effects and avoiding the negative effects of systemic medications (3, 16). Laminotomy is currently the most popular technique for DRG injection with direct access to the DRG, but this may damage a segment of the spinal nerve and the DRG (17). Furthermore, due to the absence of vertebral plate, the impact on spinal motor function and the consequent secondary injuries are inevitable (18).

Here, we describe an easy and rapid microinjection technique that enables direct injection of the agents into the DRG under direct observation. We remove the inferior articular eminence of the upper conus and the superior articular eminence of the next conus at the injection site, determine the minimum extent and best execution of the partial bone removal approach, and directly expose the DRG at the target location for local microinjection. At the same time, we tested the stability of this injection approach using dye and viral staining, the DRG could be targeted without leaking. Additionally, we observed that blocking DRG neurons substantially reversed the SNI mice’s decreased pain threshold. Our study revealed a safe and effective microinjection method that, combined with neuroscience techniques, may provide a new intervention for the clinical treatment of neurological disorders.

## Materials and methods

### Animal subjects

Male C57 (6–8 weeks, 20–25 g) mice were supplied from Tongji Hospital, Tongji Medical College, Huazhong University of Science and Technology, Wuhan, Hubei, China. All animals were raised under controlled conditions (22–25°C, 12-h alternate circadian rhythm, free access to food and water, 3–4 mice per cage). All experiments received approval from the Experimental Animal Care and Use Committee of Tongji Medical College, Huazhong University of Science and Technology, and were in agreement with the National Institutes of Health Guidelines for the Care and Use of Laboratory Animals.

### Injection

**Figure 2:**
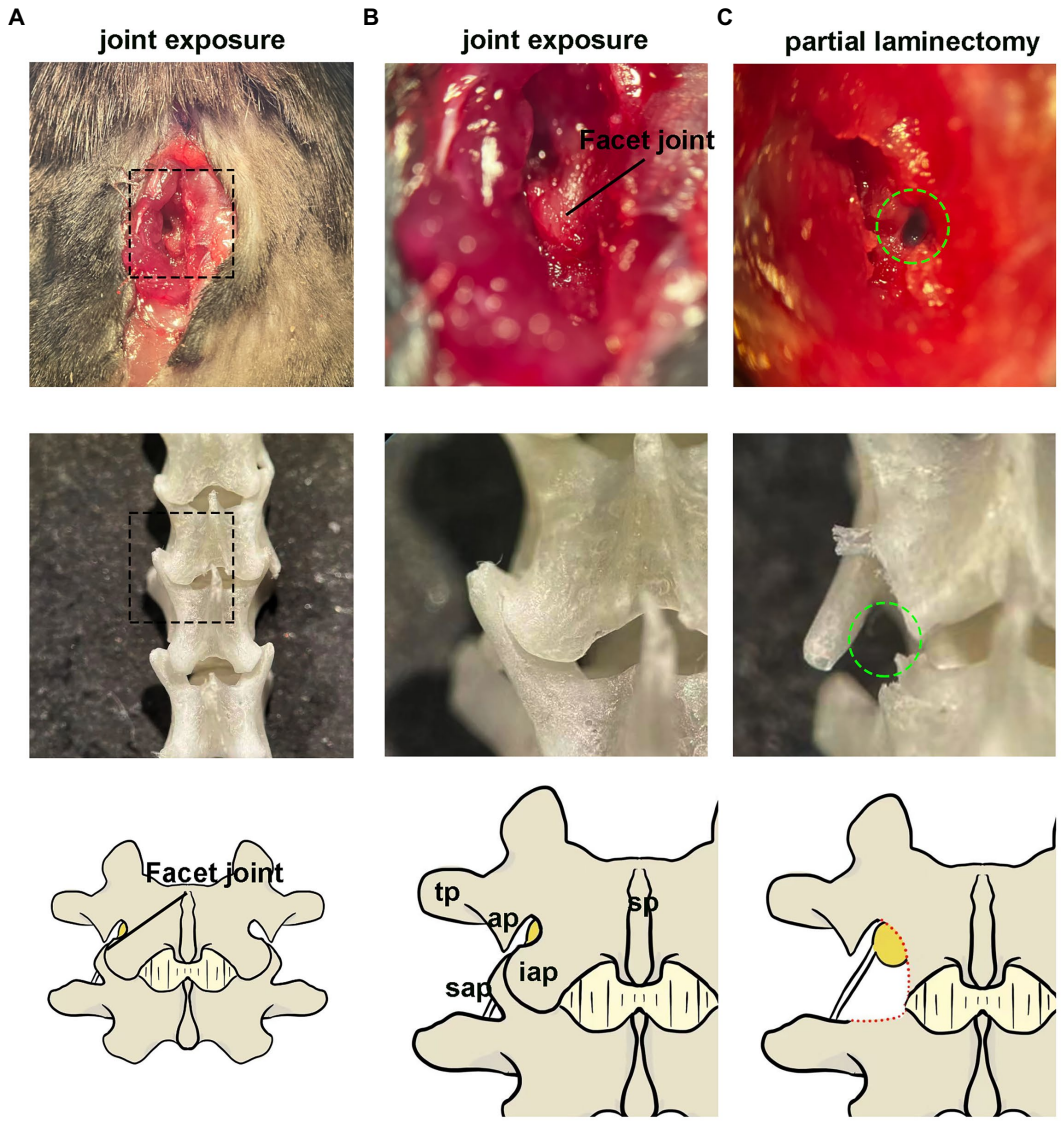
Paravertebral surgical exposure for ganglionic injection. Images show as the operative field (top panel), cleaned vertebral bones (middle panel), and reference diagram (bottom panel). **(A,B)** Preliminary soft tissue dissection at the level of the L4–L5 facet joint shows the spinous processes (sp), inferior articular process(iap), and superior articular processes (sap), as well as the laminar bone (lam) and accessory process(ap) on L4. The dorsal root ganglia are covered by a facet joint. **(C)** Removal of the facet joint bone above the foramen reveals the distal dorsal ganglion, which is much larger in diameter and located under the arm of L4 accessory process.

The DRG injection was performed using a prone position anesthetized with pentobarbital (Figure 2). The skin was shaved and disinfected, and a longitudinal, approximately 1 cm incision was made over the middle above the posterior superior iliac spine to expose the subcutaneous muscles (Figure 2A). Identify the first junction of the spinous process and silvery white aponeurosis as the location marker (L1 spinous process) [Figure 2C. A muscle incision is made immediately adjacent to the spinous process on the left side and the paravertebral muscles are bluntly separated to expose the cone, where the left intervertebral joint and left vertebral plate are visible (Figures 2D–G)]. The DRG is located below the intervertebral joint when the mouse is in prone position. The inferior articular eminence was amputated with a cranial drill and ophthalmic scissors along the line from the lumbar to the inferior edge of the previous cone (Figure 2H), and the superior articular eminence of the next cone was intercepted in a horizontal direction (Figure 2I). The accessory process is visible after the osteotomy, and the DRG is in its axilla (Figures 2J–L). The surrounding connective tissue is carefully and bluntly separated.

All injections were performed using a microprocessor-controlled injection system employing a stereotaxic holder (Item: 68030, RWD, Shenzhen, China), equipped with a pulled glass capillary injection tip (Figures 2M,N). Fine tune the needle path until the needle lumen was aligned with the center of the DRG and moved vertically downward until the tip begins to retract into the DRG. The tip was advanced further in small increments until the tip pierced the DRG (the surface depression of the DRG was restored at the moment). Slowly and gradually withdraw so that the tip was submersed in the three-dimensional center of the DRG. A quantity of agent (dye, saline or AAV) was injected through a calibrated glass microelectrode connected to an infusion pump (Hamilton CMA 400) at a rate of 20 nl/s. At the end of the injection, the pipette was left at the injection site for 5 min to avoid virus. Finally, the incision was sutured and disinfected with iodophor.

### Induction of neuropathic pain

The neuropathic pain was performed according to the procedures previously described (19). After a mouse was given a pentobarbital sodium anesthetic (50 mg/kg, intraperitoneally), the left sciatic nerve, which has three branches (the common peroneal, tibial, and sural nerves), was visible. The common peroneal nerve and the tibial nerve, the two branches of the sciatic nerve, were sectioned distally to the ligation and tightly bound with a 5.0 silk thread (or suture), removing 2–4 mm of the distal nerve stump. The intact sural nerve was carefully avoided by preventing any contact or tension. The skin was then shut.

### Basso-Beattie-Bresnahan motor function scores

Basso-Beattie-Bresnahan score was used to evaluate hindlimb motor function (20). Mice were placed on a circular platform with a diameter of 2 m. Scores for hindlimb walking and limb activity were calculated and evaluated. In the initial stage, the hindlimb joint activity received a score (0–7 points). The second stage (8–13 points) evaluated gait and coordination of the hindlimbs. The third level (14–21 points) assessed the minute paw motions made while moving. The three phases combined for a total score of 21. We performed the Basso-Beattie-Bresnahan (BBB) on day 0, 1, 3, 7, 14, and 21 after surgery.

### Punctate mechanical stimulation (von Frey)

The mechanical paw withdrawal threshold (MPWT) of the ipsilateral hind paw was determined using Von Frey filament, which simulated the mechanical allodynia as previously described (21). All behavioral evaluations were performed from 8: 30 am to 4:30 pm. Briefly, mice were placed into individual plastic containers on a metal mesh floor and acclimated to their environment for 30 min. Positive responses included shaking, licking, and rapid paw withdrawal. The minimum amount of force required to elicit a positive response or MPWT was assessed as previously discussed (in grams). An independent researcher who was unaware of the study design performed all behavioral tests.

### Open-field test

The open-field test (OFT) was used to evaluate the exploratory locomotor ability of mice. Mice were placed in the center of a grey polyethylene box (50 × 50 × 40 cm) and allowed to explore freely for 5 min. An automatic video tracking system (AVTAS v3.3; AniLab Software and Instruments Co., Ltd., Ningbo, China). Between each test, the surface of the arena was cleaned with 75% alcohol to avoid the appearance of olfactory cues.

### Immunofluorescence staining

The spinal cord and DRG tissues were taken after sodium pentobarbital anesthesia (50 mg/kg, intraperitoneal) in mice, which perfused intracardially with saline followed by 4% ice-cold paraformaldehyde in 0.1 M phosphate-buffered saline (22). The samples were then dehydrated in a 30% sucrose solution overnight at 4°C after being postfixed in 4 percent paraformaldehyde for 4 h at 4°C. Using a cryostat microtome (Thermo Fisher, NX50, Waltham, MA), the fixed spinal cord was cut into 20 μm thick coronal slices. Sections were stained for 10 min at room temperature with DAPI, followed by a PBS wash (three times, 10 min each). Finally, a virtual microscope slide scanning system (Olympus, *VS* 120, Tokyo, Japan) was used to see the immunostained sections. Photographs of sections containing the region of interest (ROI) were cropped in ImageJ (National Institutes of Health, Bethesda, MD). The quantity of cells and neurons in the DRG were measured with ImageJ. The DAPI stained nucleus was counted as total cells.

### Virus injections and chemogenetics

Briefly, mice were anesthetized with sodium pentobarbital anesthesia (50 mg/kg, intraperitoneal). For cell-type-specific *in situ* labeling, 200 nl rAAV-EF1α-EGFP-WPRE-pA was injected with the L4 DRG and detected by expression staining 3 weeks after AAV expression. For chemical inhibition, we used the virus rAAV-hSyn-hM4D (Gi)-mCherry to inhibit neuronal activity in the DRG. Mice were injected 200 nl of rAAV-hSyn-hM4D(Gi)-mCherry into the DRG. SNI surgery was performed 7 days after AAV virus injection. 14 days later, mice were given von Frey tests before and 1 h after CNO (Clozapine N-oxide, a human muscarinic designed receptor agonist; 5 mg/kg) administration intraperitoneally. The MPWT was measured 1 day after the CNO injection. Then, mice were perfused to examine viral infection in the DRG.

### Experimental designs treatment

**Figure 3:**
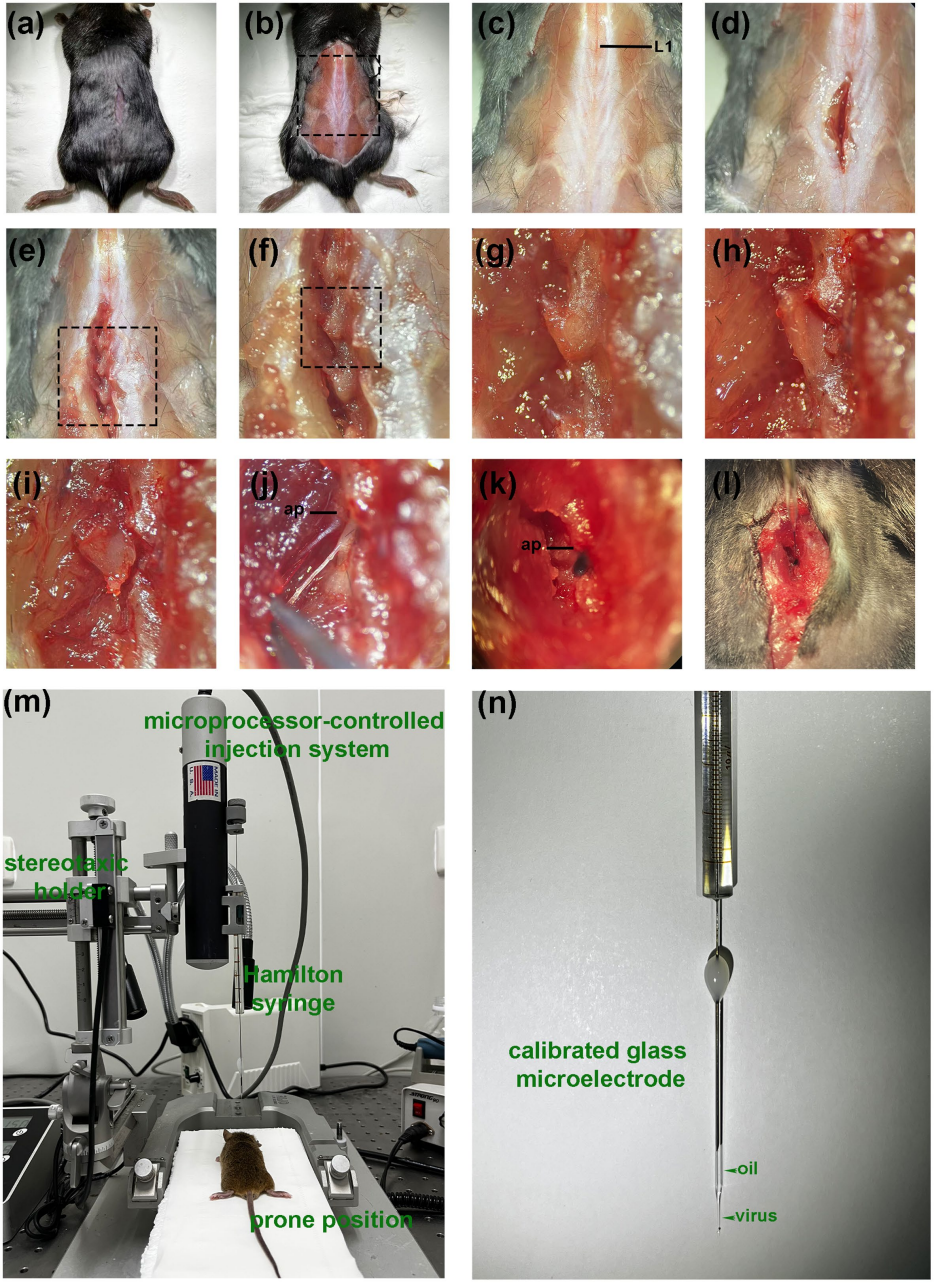
Procedure of DRG injection under direct vision. **(A)** After anesthesia, a small incision is made in the dorsal skin at the level superior to posterior superior iliac spine. **(B)** The incision is enlarged for better visualization (for display only, not part of the operation steps). **(C)** The first junction of the spinous process and the silvery white aponeurosis is identified as a positional marker (L1 spinous process). **(D)** A deep muscle incision is made along the lateral surface of the spinous process. **(E–G)** Expose the left intervertebral joint and the left vertebral plate. **(H)** Excision of the inferior articular eminence. **(I)** Excision of the superior articular eminence. **(J)** Exposure of the DRG. (The distal end is fixed with forceps because the display causes the wound to widen, resulting in distal fixation failure). **(K,L)** Visual field during in vivo injection. (The DRG is filled with dye). **(M)** Diagram of the experimental equipment set-up. **(N)** Injection using a glass needle containing mineral oil and a trace of viral solution. ap, accessory process.

**Figure 4:**
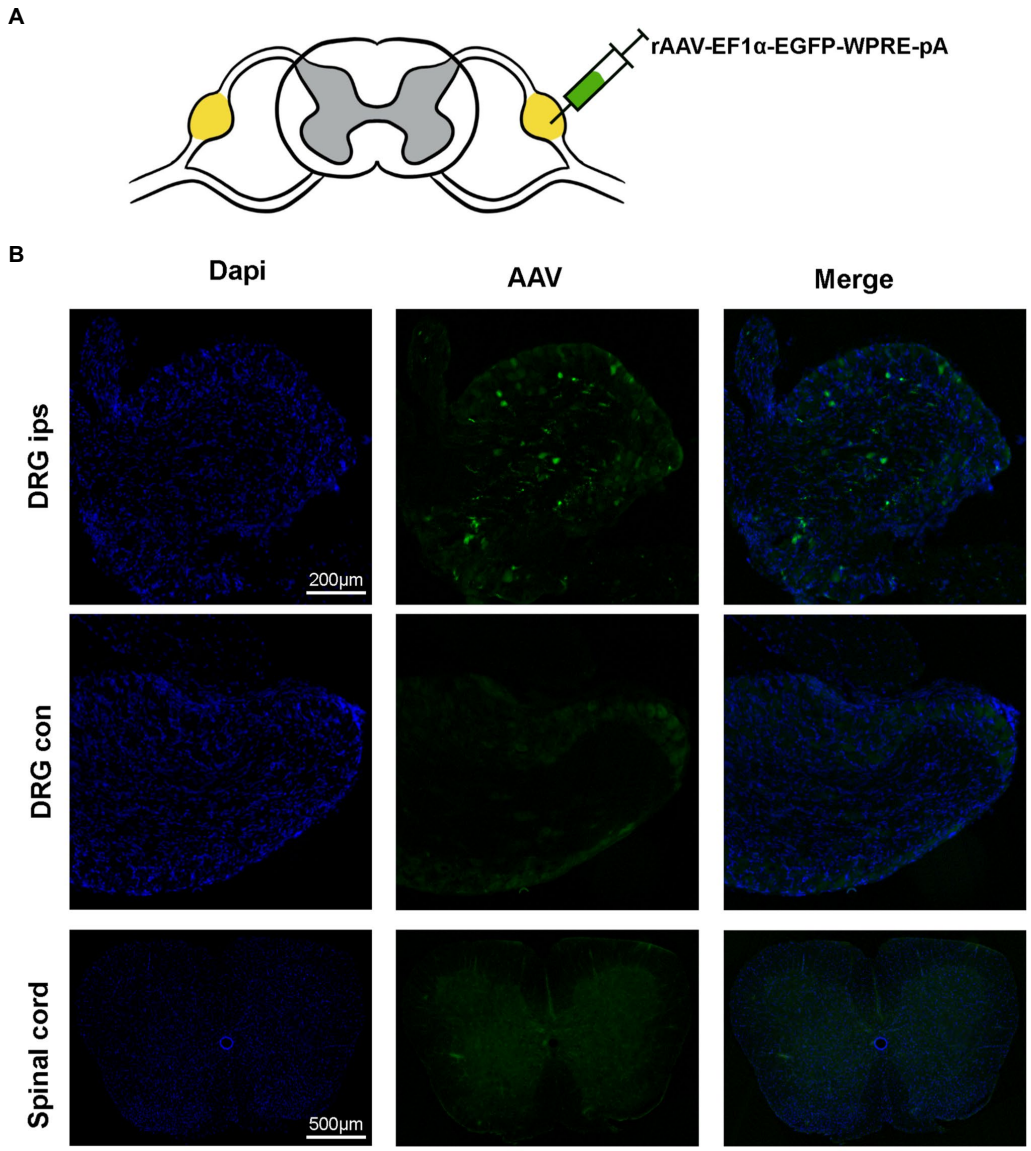
Virus expression 21 days after the DRG injection. **(A)** AAV is injected at the L4 DRG. **(B)** The virus is expressed at the injection site, in the contralateral DRG and in the spinal cord. The virus was confined to the DRG without leakage (*n* = 5 mice per group).

**Figure 5:**
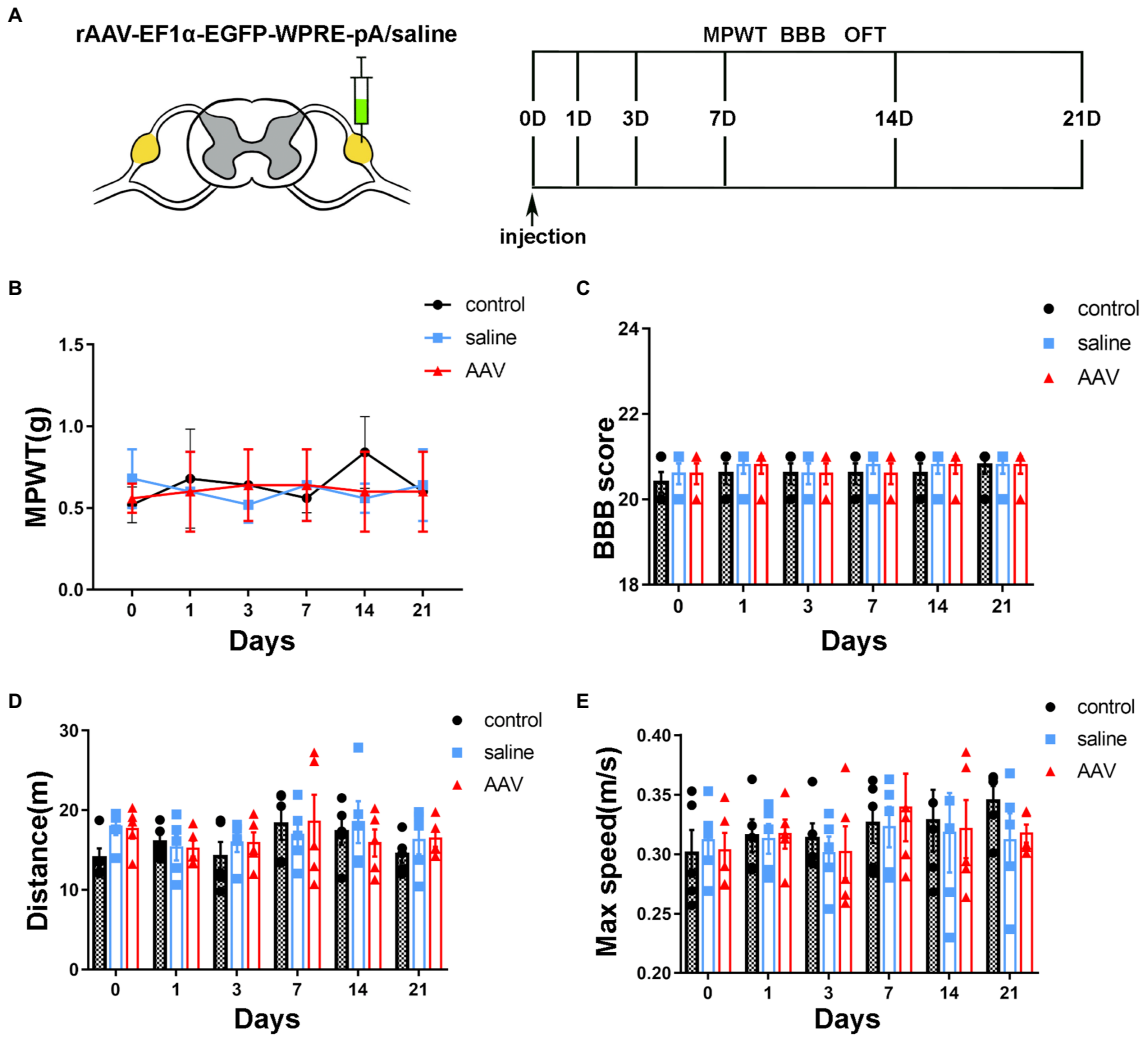
Changes in sensory and motor function after surgery and injection. **(A)** The schedule of administrating AAV or saline, BBB test, MPWT and OFT. **(B)** Compared to the control group, no effect is observed on the sensory function after saline or AAV administration (*p* > 0.05, *n* = 5 mice per group). **(C)** The scores of the control, saline and AAV groups are similar at the corresponding time points (*p* > 0.05, *n* = 5 mice per group). The manipulation had no effect on motor function. **(D,E)** In the OFT, there are no significant differences in the distance and maximum velocity among the three groups (*p* > 0.05, *n* = 5 mice per group).

The design of this study is shown in Figures 3A, 4A, 5A. First, we examined the intraoperative progress of the DRG injection with a non-toxic dye. Then, AAV was used to determine the success of injection. Meanwhile, three dosing regimens were also designed for the control, saline (200 nl), and AAV (200 nl) groups to explore the effects of the injection operation on movement and sensation. Finally, chemogenetic approach was used to inhibit DRG neurons (23). CNO (Sigma-Aldrich, dissolved in 0.1% DMSO in saline 5 mg/kg) or saline was injected intraperitoneally after 3 weeks of injection of AAV-hM4Di at the end of the virus incubation period.

### Statistical analysis

All results are shown as mean ± SEM. In analyses, an unpaired Student’s *t*-test was applied for comparing two groups. Two-way ANOVA (Analysis of Variance) was used for group comparisons, followed by the Bonferroni *post hoc* test. Applying Pearson coefficients allowed us to statistically convey relevance. GraphPad Prism 7.0 was used for statistical analysis, and *p* < 0.05 was considered statistically significant in this study.

## Results

### Partial bone removal allowed good exposure of the DRG and injection

**Figure 6:**
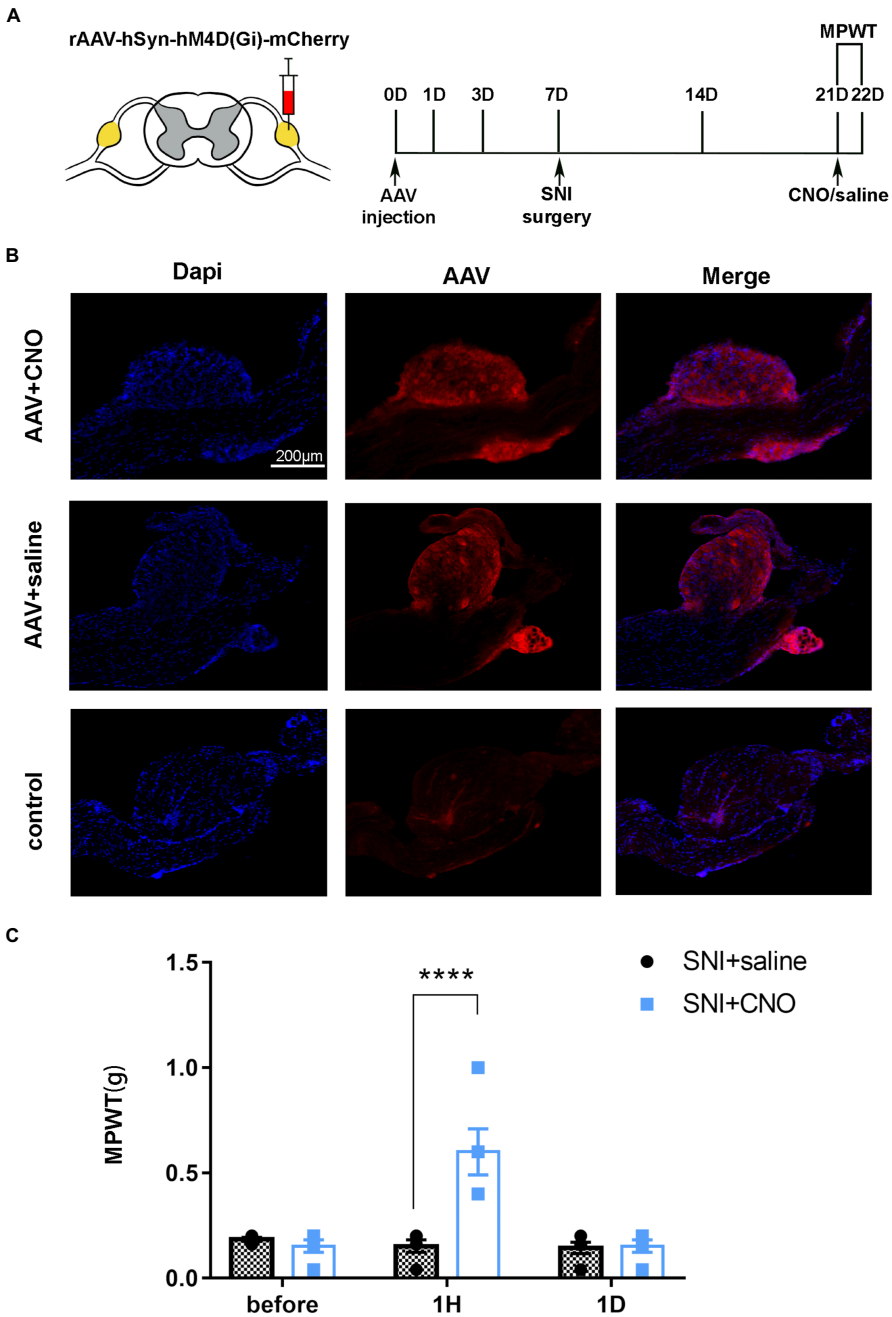
DRG may serve as a target for chemogenetic reversal allodynia induced by SNI. **(A)** The schedule of administrating AAV, saline or CNO, MPWT. **(B)** AAV virus expressed in the DRG (*n* = 5 mice per group). **(C)** Compared to the saline group, CNO effect reverse allodynia induced by SNI (****p < 0.0001, *n* = 5 mice per group), with effect disappearing after 1 day (*p* > 0.05, *n* = 5 mice per group).

The anatomical location of the lumbar lateral dorsal root ganglion is under the facet joint and under the transverse axilla when the mice were in prone position (Figure 1). The DRG was exposed by removing the upper and lower articular processes corresponding to the target DRG, as shown in Figure 6. The DRG under the transverse axillary fossa after partial osteotomy could be seen directly through the microscope (Figure 2). The drawn glass capillary was adjusted above the target DRG, and the height of the capillary was adjusted vertically down through the DRG and successfully submerged under direct vision. Afterwards, 1 μl of dye was injected directly into the DRG, which showed no extra-DRG dye leakage and no DRG rupture or damage under microscopy (Figures 2H,I).

### Conventional doses of virus were injected into the DRG without leakage

To detect the modulation of neuronal function in the target DRG using this method can be achieved with less damage. We injected AAV virus into the target DRG and tested the viral expression after 3 weeks of viral transfection (Figure 3A). The results of immunofluorescence showed that the virus was expressed only in the ipsilateral DRG and did not spread to the contralateral and spinal cord (Figure 3B). And the rate of positive cells, as a percentage of all cells, is 2.3516233% ± 0.3783797% (*n* = 3 mice).

### Motor and sensory functions were not affected after virus injection into the DRG of mice

mechanical paw withdrawal threshold was used to detect mechanical allodynia. BBB and FCT were performed to evaluate the motor function after virus injection. The behavioral tests were performed on days 0, 1, 3, 7, 14, and 21 (Figure 4A). Compared to the control group, there was no significant difference in MPWT after injection of saline or AAV (Figure 4B, group: *F*(2, 72) = 0.2632, *p* = 0.7694; time: *F*(5, 72) = 0.2695, *p* = 0.9284; interaction: *F*(10, 72) = 0.8821, *p* = 0.5538). BBB score showed that there was no decrease in motor ability of hind limb after the operation (Figure 4C, group: *F*(2, 72) = 0.5652, *p* = 0.5707; time: *F*(5, 72) = 0.5652, *p* = 0.7263; interaction: *F*(10, 72) = 0.09565, *p* = 0.9998). In OFT, no significant difference was found in the distance (Figure 4D, group: *F*(2, 72) = 0.741, *p* = 0.4802; time: *F*(5, 72) =1.121, *p* = 0.3573; interaction: *F*(10, 72) = 0.5286, *p* = 0.8645) and max speed (Figure 4E, group: *F*(2, 72) = 0.2902, *p* = 0.7490; time: *F*(5, 72) = 0.8515, *p* = 0.5180; interaction: *F*(10, 72) = 0.2137, *p* = 0.9943) among the control, saline and AAV groups. These results suggest that there was no dysfunction of motor or sensory after injection in long or short periods.

### Application of chemical inhibitors in DRG effectively inhibited hyperalgesia induced by SNI

A pharmacologic inhibitor of the virus CNO was used to test whether selective inhibition of mice DRG neurons suppressed SNI-induced behavioral responses (Figure 5A). The results of immunofluorescence showed that AAV-hM4Di injection was expressed in the DRG and the rate of positive cells is 3.8515644% ± 1.8687406% (Figures 5B, *n* = 3 mice). Notably, the mechanical allodynia induced by SNI was reversed on day 14 after administration of CNO (Figures 5C, *t* = 6.248, *P* < 0.0001). However, compare to the saline group, there was no significant difference in MPWT 1 day after administration of CNO (Figures 5C, *t* = 0.1116, *p* > 0.9999). These results further support that local injection of DRG modulated the development of chronic pain.

## Discussion

The present study has defined a method for selective injection of drugs or viruses into the target DRG. Our research achieved a new minimally invasive and intuitive intra-ganglionic injection in mice. Intra-DRG injection does not cause sensory or motor function. In addition, injection of chemical inhibitors in L4 DRG of SNI mice effectively alleviated mechanical hypersensitivity caused by surgery, which confirmed that our improved technique offers additional possibilities for studying sensory mechanisms and treating chronic neuropathic pain.

Various methods have been used to deliver agents to the DRG for application study (24). Although the basic method of alternative injection sites for DRG treatment, intrathecal injection by lumbar puncture, is simple to perform, agent diffusion is unavoidable due to the high doses of medications utilized (25, 26). In contrast to direct injection into the DRG, sciatic nerve injection harms peripheral nerves and targets numerous DRGs (27). Due to the severe vertebra impairments, direct injection laminectomy causes spinal cord instability and sensory and motor dysfunction (28). Partial laminectomy mentioned by Gregory Fischer requires a shallow angle of incidence to the nerve surface (29). Our research had several benefits for the delivery of agents to the DRG. First, injection is carried out utilizing a Hamilton syringe and a Small Animal Stereotactic Frame with the Microinjection Adaptor, potentially causing less damage to the DRG and offering a solid platform for injection. Second, a moderate amount of bone removal maintains the spine’s stiffness, allowing to better preserve neurological function and improving the accuracy and repeatability of following studies by preventing further damage from spinal instability in the late postoperative period. Thirdly, after removal of the articular eminence, the DRG is located in the axilla of the anapophysis which attached to a silver-white tendon. This simple dissection procedure can be easily completed in 20–30 min per mice due to the clear positioning markings, from animal anesthesia to injection. Finally, there is no need to adjust the angle of injection, and direct vision under the microscope reduces the risk of missing the target and completely penetrate the DRG and reach the ventral root. Meanwhile, small incisions were required under direct vision to avoid hyperalgesia induced by surgical exposure of the ganglion.

DRG-targeted injections may serve as an important means of modulating sensory function (2), so it is critical to ascertain the effect of the injection. In contrast to the control group, our data showed that there was no hyperalgesia following surgical exposure and ganglion injection. DRG segmental injection has a potential future in the field of viral vector-mediated gene therapy. Our findings demonstrated that by directing DRG injections to modulate neuronal activity, significant transduction can be accomplished. The use of neuron-specific viruses in our study could achieve an infection rate (The proportion of positive cells in the total number of cells) of approximately 2% (rAAV-EF1α-EGFP-WPRE-pA) and 4% (rAAV-hSyn-hM4D(Gi)-mCherry). Mecklenburg ‘s research estimated that the overall percentage of sensory neurons among all mice DRG cells is about 3–5% (30, 31). And in the current study, we discovered that in SNI rats, inhibiting the DRG neurons can significantly reduce mechanical allodynia. The mechanical allodynia caused by SNI was shown to be effectively reduced by inhibiting the DRG neuronal activity, but the relief was only temporary. This may be because CNO binds to hM4Di to hyperpolarize the cell membrane and inhibit the action potential of neurons, and CNO typically takes 2 h to clear from the membrane after being applied to the neuron. (32). Thus, by combining anatomically selective drug delivery mechanisms with novel molecularly selective drugs, along with other neuroscientific tools, this protocol may promote new strategies for treating chronic pain. The dissection technique described in this study does have its limitations. Firstly, the method is primarily applicable to the lumbar DRGs and is unlikely to be applied at other places. Secondly, reinjection is impossible, but the use of indwelling tubes within the DRG could be considered. Finally, the advantages of this strategy over other ways need to be further investigated because our study did not compare it to previous ones. However, this option would support preclinical studies of injectable solutions administered intralaryngeally to advance previous findings in rodent studies.

## Conclusion

Techniques for *in vivo* injection can make it easier to explain the correlation between behavior and underlying mechanism. Our research provided a quick, efficient, and visible method for single intra-DRG injection. The results of the current investigation demonstrated that no motor or sensory deficits developed throughout the injection procedure. Chemical inhibition of DRG neuron alleviated hyperpathia induced by SNI. Therefore, DRG may be a promising target for neuropathic pain researches, and *in vivo* injection of DRG under direct vision provide an effective method for the further exploration of the nervous system.

## Data availability statement

The original contributions presented in the study are included in the article/supplementary material, further inquiries can be directed to the corresponding authors.

## Ethics statement

The animal study was reviewed and approved by Experimental Animal Care and Use Committee of Tongji Medical College, Huazhong University of Science and Technology.

## Author contributions

XY conceived the research, carried out the model building, coordinated the lab work, and drafted the manuscript. SH and FZ performed the statistical analysis and drafted the manuscript. AM and FG took care of the behavior tests and drafted the manuscript. JW, WZ, and XT participated in its design and coordination and helped to draft the manuscript. All authors contributed to the article and approved the submitted version.

## Funding

This work was supported by the National Natural Science Foundation of People’s Republic of China (grant nos. 81974170) and the Natural Science Foundation of Hubei Province (grant no. 2021CFB341).

## Conflict of interest

The authors declare that the research was conducted in the absence of any commercial or financial relationships that could be construed as a potential conflict of interest.

## Publisher’s note

All claims expressed in this article are solely those of the authors and do not necessarily represent those of their affiliated organizations, or those of the publisher, the editors and the reviewers. Any product that may be evaluated in this article, or claim that may be made by its manufacturer, is not guaranteed or endorsed by the publisher.

## Abbreviations

DRG: Dorsal root ganglion
SNI: Spared nerve injury
MPWT: The mechanical paw withdrawal threshold
CNO: Clozapine-N-oxide
